# Is There a Conspiracy of Silence?

**Published:** 1914-10

**Authors:** 


### Is There a Conspiracy of Silence?

The medical profession is charged with a conspiracy of silence; it is claimed that physicians deliberately attempt to prevent the spread of their knowledge. Is the charge true?

We have never known any person to appeal to physicians for information and be refused. The average doctor will gratuitously spend his time discussing any medical problem propounded to him. The people at large have ready access to all the information they want. It has been customary to accuse all erudite individuals of airing their knowledge, and doctors are no exception. On the other hand, it is undoubtedly a fact that the average doctor is very reluctant to appear in print. There are several reasons for his attitude.

The physician is human, and does not like to waste his ammunition firing into the air. It is well known that harangues printed in the newspapers are not read as a rule. What man likes to give advice when it is not heeded? When a piece of information is printed by the lay press it is presumably addressed to the public at large. The invitation then to give this advice should also come from the public at large. Instead it comes from a newspaper reporter who may or may not know what the public desires. Moreover, when a doctor launches an article in the public press he is addressing not only his patients, who know him and believe in him, but he is addressing also the patients of all the other doctors, and the latter may not depend on or care for his advice.

Then there is much probability that the advice will miscarry and be misconstrued. The gap between the learned and the unlearned is truly a deep and wide one. Medicine is the only technical science related to the daily life of every man that lives. It is the most closely connected with our birth, life, and death. Electrical engineers are not importuned daily to publish their views on the various problems of their work, because the people are interested only casually. The same is true in the case of all the trades and professions except medicine. We physicians then are faced by a problem that no others face: we are besieged by people who desire to know the technical intricacies of our work. The difficulties are so great when we attempt to elucidate that we prefer to do it face to face. We dislike to give vent to a lot of views which are almost sure to be misunderstood and may be actively distorted. That is why the doctors prefer to refrain from publishing their private opinions.

But they have no conspiracy of silence.

